# Working with argan cake: a new etiology for hypersensitivity pneumonitis

**DOI:** 10.1186/s12890-015-0013-3

**Published:** 2015-03-06

**Authors:** Christophe Paris, Fabrice Herin, Gabriel Reboux, Emmanuelle Penven, Coralie Barrera, Cécile Guidat, Isabelle Thaon

**Affiliations:** Université de Lorraine, INGRES, EA 7298, Vandoeuvre lès Nancy, F-54505 France; CHU Nancy, Centre de consultations de pathologies professionnelles, Vandoeuvre lès Nancy, F-54511 France; CHU Toulouse, Service des Maladies Professionnelles et Environnementales, Toulouse, F-31000 France; Université de Toulouse, UMR 1027, Toulouse, F-31000 France; UMR/CNRS 6249 Chrono Environnement, Université de Franche-Comté, Besançon, F-25030 France; Service de Parasitologie-Mycologie, CHU J. Minjoz, Besançon, F-25030 France; Association Lorraine de Santé en Milieu de Travail, Pulnoy, F-54425 France

**Keywords:** Hypersensitivity pneumonitis, Argan, Occupational diseases

## Abstract

**Background:**

Argan is now used worldwide in numerous cosmetic products. Nine workers from a cosmetic factory were examined in our occupational medicine department, following the diagnosis of a case of hypersensitivity pneumonitis (HP) related to handling of argan cakes.

**Methods:**

Operators were exposed to three forms of argan (crude granulates, powder or liquid) depending on the step of the process. All workers systematically completed standardized questionnaires on occupational and medical history, followed by medical investigations, comprising, in particular, physical examination and chest X-rays, total IgE and a systematic screening for specific serum antibodies directed against the usual microbial agents of domestic and farmer’s HP and antigens derived from microbiological culture and extracts of various argan products. Subjects with episodes of flu-like syndrome several hours after handling argan cakes, were submitted to a one-hour challenge to argan cakes followed by physical examination, determination of Carbon Monoxide Diffusing Capacity (DLCO) and chest CT-scan on day 2, and, when necessary, bronchoalveolar lavage on day 4.

**Results:**

Six of the nine workers experienced flu-like symptoms within 8 hours after argan handling. After challenge, two subjects presented a significant decrease of DLCO and alveolitis with mild lymphocytosis, and one presented ground glass opacities. These two patients and another patient presented significant arcs to both granulates and non-sterile powder. No reactivity was observed to sterile argan finished product, antigens derived from argan cultures (various species of *Bacillus)* and *Streptomyces marokkonensis* (reported in the literature to contaminate argan roots).

**Conclusions:**

We report the first evidence of hypersensitivity pneumonitis related to argan powder in two patients. This implies preventive measures to reduce their exposure and clinical survey to diagnose early symptoms. As exposure routes are different and antibodies were observed against argan powder and not the sterile form, consumers using argan-based cosmetics should not be concerned.

**Electronic supplementary material:**

The online version of this article (doi:10.1186/s12890-015-0013-3) contains supplementary material, which is available to authorized users.

## Background

Argan is now used worldwide in numerous cosmetic products, as this fruit is supposed to possess many health properties related to its high unsaturated fatty acid and phytosterol content [1,2]. Argan oil is obtained according to an ancestral technique by heating, roasting and pressing the nuts contained in the fruit of *Argania spinosa* (Sapotaceae), an endemic tree growing in arid and semi-arid areas in South-West Morocco.

More recently, argan-derived products (proteins) extracted from argan cakes (residuals from pressing operations) have also been used in cosmetic products. To date, only one case of argan oil allergy has been reported in the literature, with digestive manifestations related to argan oil ingestion [3].

In January 2011, a 35 year-old man, a current smoker working in a cosmetic factory, was referred to the occupational medicine department of our university hospital. He reported brief episodes of flu-like syndrome (asthenia, myalgia, shivering, chest tightness) several hours after handling of crude argan powder derived from argan cakes. With his written and informed consent, the patient underwent a specific challenge to argan cake milling products at his workplace for one hour (D0), under medical supervision. This challenge was followed, eight hours later, by flu-like syndrome. Diffusing capacity of the lung for carbon monoxide (DLCO) decreased from 93% predicted to 80% at D2 with no changes on HRCT scan. On D4, alveolitis (602,000 cells/mL) with discrete lymphocytosis (25%), predominantly CD8 (60%), was observed in bronchoalveolar lavage (BAL). Specific serum antibodies directed against antigens derived from extracts of various argan products were positive with up to seven arcs on electrosyneresis (ES). The diagnosis of Hypersensitivity Pneumonitis (HP) was established in this patient according to form 1 of the HP study classification [4].

Following this index case, presenting a new etiology for HP, we decided to conduct a systematic survey of all workers exposed under similar conditions in order to identify other possible cases of HP. A second objective was to identify the antigen responsible (microorganisms as in farmer’s lung or protein as in pigeon breeders’ lung [5]).

## Methods

### Description of the industrial process

Argan cakes (as granulates) arrive from Morocco in jute bags of 25 kg with no treatment after the pressing step. Argan cakes are initially delipidized by carbon dioxide at low temperature, and then milled to a fine powder. A second step of the process, including solubilization, filtration and sterilizing operations, results in a sterile finished product, in powder or liquid form. Operators are exposed to various levels of three forms of the product (granulates, powder and sterile powder) depending on the step of the process and protective measures. Workers wear filtration masks only during one step.

### Data collection

All subjects were examined in our reference center after specific information on the examinations and after obtaining their written informed consent. All patients filled in a standardized questionnaire including personal use of argan products (in cosmetics or cooking), personal history of atopy, allergic rhinitis or asthma, smoking, flu-like symptoms (asthenia, myalgia, fever, chest tightness) after argan handling. Physical examination, anteroposterior chest X-ray and blood tests, including total IgE, were performed in the absence of any recent argan exposure. After specific treatment and extraction of proteins from argan products (see below), a specific identification of precipitins in workers’ sera was systematically performed.

All subjects with episodes of flu-like syndrome several hours after handling argan cakes, underwent the same workplace specific challenge test to argan powder as that performed in the index case. The challenge test was organized in the workplace with the collaboration of the factory occupational physician and a resident of our center, under the usual conditions of the industrial process. On D2, the workers underwent physical examination with blood count, of pulmonary function tests by plethysmography (Vmax - Autobox V62J Encore, SensorMedics, Yorba Linda, CA), and chest HRCT scan. On D4, BAL was proposed only in subjects with flu-like symptoms during the hours following argan exposure and association of significant features on either HRCT scan (ground-glass opacities) or pulmonary function tests (more than 10% decrease DLCO after challenge).

### Culture and antigen extracts

The three forms of argan were processed by one of the authors (GR) to extract microorganisms and specific antigens. The methods have been previously published [6,7]. Endotoxin assay was also performed by the National Institute of Safety Research (INRS) [8]. Culture and isolations methods are described in Additional file 1.

Three total crude extracts were prepared by extraction from crude sample materials according to the form: granulates, powder and sterilized finished product. This technique is adapted from Pépys *et al*. [9] (see Additional file 1). Two somatic antigens (*Bacillus Licheniformis* and *Bacillus* sp.) were derived from bacteria isolated from “argan” (powder and granulates). Antigens were prepared as previously described [7] (see Additional file 1).

As a review of the literature showed that argan roots are commonly contaminated by *Streptomyces marokkonensis* [10], *S. marokkonensis* antigen was prepared from the original strain (DMZ 41918) isolated from the *Argania spinosa* tree.

For ELISA technique, special protein form of antigens was made using a recombinant β-1,3-glucanase and proteins were pelleted with trichloroacetic acid [11] (see Additional file 1).

### Serology techniques

All sera were tested by two techniques using the same batches of antigen. Serum precipitins were investigated by agar gel Ouchterlony double diffusion (DD) [12] and electrosyneresis (ES) on cellulose acetate [13]. All results were read blindly by two operators and no difference was observed between the two readings. Serological techniques have been previously described in detail in supplementary data on Journal of Medical Microbiology [6]. Moreover an ELISA IgG technique (Enzyme-Linked ImmunoSorbent Assays) was also used for all sera. Technique was described previously by Roussel *et al.* [11].

## Results

Nine subjects, one female and eight male, including the case index, were included in the study. Their main characteristics are reported in Table 1. Six subjects, including the index case, reported similar respiratory symptoms: flu like symptoms early in the evening following argan handling. Physical examination and initial chest X-rays in the absence of any recent exposure to argan were normal in all patients (Table 2). Table 2 also presents the results of specific challenge tests.

**Table 1:** **General and occupational characteristics of the subjects sample**

**Cases**	**Gender (age)**	**Tobacco status**	**Domestic argan exposure**	**Workstation**	**First exposure to argan**	**Frequency of exposure to argan granule**	**Last exposure to argan**	**Complaints suggestive of HP**	**Onset and duration of complaints suggestive of HP**
1	Male (35)	S	No	production operator	2006	monthly	previous month	flu-like syndrome	Early evening and for several hours
2	Male (28)	FS	No	production operator	2008	monthly	previous month	flu-like syndrome	Early evening and for several hours
3	Male (35)	FS	No	production operator	2007	monthly	previous month	flu-like syndrome	Early evening and for several hours
4	Male (39)	FS	No	production (foreman)	2007	monthly	previous month	flu-like syndrome	Early evening and for several hours
5	Male (30)	S	No	production operator	2007	occasionally	previous month	No	/
6	Male (35)	S	No	production operator	2007	monthly	previous month	No	/
7	Male (45)	S	No	“powder” workshop	2006	occasionally	previous month	No	/
8	Female (40)	NS	No	packaging	2007	monthly	previous month	No	/
9	Male (34)	NS	No	production operator	2006	occasionally	previous month	flu-like syndrome	Early evening and for several hours

S = Smoker; FS = Former smoker; NS = Non smoker.

Flu-like syndrome: myalgia, asthenia, fever, chest tightness.

**Table 2:** **Summary of investigations for the diagnosis of Hypersensitivity pneumonitis**

**Before specific challenge**				After specific challenge *				
**Cases**	**Physical examination**	**Chest X rays**	**DLCO**	**Symptoms suggestive of HP** ^**†**^	**DLCO (variation) % predicted** ^**†**^	**CT Scan** ^**†**^	**BAL** ^**‡**^	**Conclusion**
1	normal	normal	89%	Yes	78% (-11%)	No specific abnormalities	600000/mL 25.5% lymphocytes	HP
2	normal	normal	118%	Yes	120%	/	/	ODTS
3	normal	normal	63%	Yes	61%	Emphysema	935000/mL 5% lymphocytes	ODTS
4	normal	normal	88%	Yes	62% (-22%)	Localized Ground-glass opacities	600000/mL 18% lymphocytes (not available)	HP
5	normal	normal	68%	Yes	79%	/	/	ODTS
6	normal	normal	85%	/	/	/	/	/
7	normal	normal	79%	/	/	/	/	/
8	normal	normal	86%	/	/	/	/	/
9	normal	normal	95%	Yes	94%	/	/	ODTS

* one-hour challenge (D0) to argan cakes at the workplace, only performed in the 6 subjects with flu-like symptoms in the 8 hours following argan handling.

^†^ at day 2 after challenge.

^‡^ at day 4 after challenge-, only performed in subjects with significant features either on HRCT scan (ground-glass opacities) or pulmonary function tests (more than 10% decrease of DLCO after challenge).

/: not performed.

HP: Hypersensitivity Pneumonitis, ODTS: Organic Dust Toxic Syndrome.

In the index case (#1), the challenge reproduced the symptoms within 8 hours, but physical examination remained normal. HRCT scan did not demonstrate any significant abnormalities. BAL revealed the presence of alveolitis with 602,000 cells/mL and discrete (25%) but significant lymphocytosis in this smoker [14,15] with a predominance of CD8 (60%). Noteworthy, the DLCO was decreased to 78% predicted.

Three of the five new symptomatic patients (#2, #5 and #9) did not present any alteration of DLCO or HRCT scan after specific challenge. In subject #3, a current smoker, who presented an abnormal DLCO before and after the challenge (63% and 61% predicted, respectively), HRCT scan demonstrated the presence of centrilobular emphysema. BAL was at the limit of normal. The last subject (#4), also a current smoker, presented a significant decrease of DLCO after the challenge (62% predicted *vs* 88% predicted), and localized ground-glass opacities on HRCT scan on D2 after challenge. However, BAL failed to demonstrate the presence of lymphocytosis in this case (18%). As this stage, the diagnosis of HP was only suspected in this subject.

### Microbiological results

Only two *Bacillus* species were identified in all aqueous samples: *Bacillus licheniformis* and *Bacillus* spp. 200 CFU/g and 400 CFU/g of *B. licheniformis* in granulated and “argan” powder, respectively. *Bacillus* spp. was isolated at a concentration of 400 CFU/g and 2000 CFU/g in the same samples. In a second sample of argan cake, 3 strains of *Bacillus* were identified by DNAr 16S sequence and phenotypic characteristics (*B. pumilus, B. clausii, B. subtilis*). No other bacteria, actinomycetes or fungi were isolated.

### Serology results

The two patients (#1 and #4) with suspected HP after the specific challenge presented significant arcs to both granulates and non-sterile powder (seven and three arcs with ES technique in case #1 and five and three arcs in case #4, respectively, see Table 3). Patient #9, who reported flu-like symptoms after argan handling but with no change in DLCO or HRCT scan after specific challenge, also presented significant arcs to both granulates and non-sterile powder (four and three arcs with ES technique, respectively). These three patients also had elevated IgG against argan granulates and/or argan powder (Table 4).

**Table 3:** **Number of precipitin arcs for four antigens in workers exposed to argan and non-exposed controls**

**Case**	**Precipitin arcs n**									
	**Argan granulates**		**Argan powder**		**Sterile finished product**		***Bacillus*** **spp**		***Streptomyces marokkonensis***	
	ES*	**DD** ^**†**^	**ES**	**DD**	**ES**	**DD**	**ES**	**DD**	**ES**	**DD**
**Workers exposed to Argan**										
1	7	4	3	4	1	1	0	1	0	0
2	2	0	0	2	0	0	0	0	1	0
3	2	1	1	1	0	0	0	2	0	0
4	5	2	3	2	0	0	0	1	1	0
5	2	1	1	1	0	0	0	1	1	0
6	2	1	1	1	0	0	0	0	1	0
7	2	1	1	1	0	0	0	1	0	0
8	2	1	1	1	0	0	0	0	1	0
9	4	3	3	1	0	0	0	0	0	0
**Non exposed Controls**										
1 (serum pool^‡^)	1	0	0	2	0	0	0	0	0	0

*ES = electrosyneresis.

^†^double diffusion.

^‡^from 9 other urban adults without exposure to farm products or birds.

**Table 4:** **Mean IgG and total IgE in workers exposed to argan and exposed controls**

**Case**	**Total IgE (IU/ml)**	**Average IgG**				
		**Argan granule Ag (ARG Ag)**	**Argan powder Ag (ARP Ag)**	**Argantensyl Ag (ART Ag)**	***Bacillus*** **spp. Ag**	***Streptomyces. marokkonensis*** **Ag**
**Workers exposed to Argan**						
1	80	2.48	2.37	3.25	0.13	0.10
2	559	1.10	1.00	0.55	0.12	0.13
3	261	1.17	0.94	0.22	0.10	0.29
4	144	0.96	1.48	1.48	0.10	0.14
5	100	1.30	1.03	0.27	0.10	0.19
6	47	1.52	0.92	0.45	0.11	0.09
7	21	0.14	0.22	0.14	0.13	0.09
8	65	0.51	0.56	0.28	0.10	0.10
9	112	1.49	1.25	1.12	0.13	0.09
**Non exposed controls**						
1 (serum pool*)	/	0.45	0.62	0.22	0.14	0.11

*from 9 other urban adults without exposure to farm products or birds.

No reactivity was observed against antigens derived from bacteria (*Bacillus* spp. including *B. pumilus, B. clausii, B. subtilis, B. licheniformis* and *S. marokkonensis*) in any of the subjects. Noteworthy, none of the subjects presented arcs against the sterile argan finished product. No arcs were identified in a serum pool of 9 urban adults without exposure to farm products or birds. None of subjects presented any exposure to other common risk of HP.

Finally, on the basis of the presence of clinical symptoms after exposure, ground-glass opacities on HRCT scan and positive serology, the diagnosis of HP was adopted for case #4 as well as case #1.

## Discussion

Two cases of documented HP related to handling argan cake were identified among nine exposed workers in a factory using argan cake. Positive diagnosis was based on the presence of the following criteria after specific challenge test at the workplace: presence of suggestive clinical symptoms within 8 hours, significant specific serum antibodies to extracts or argan granulates and non-sterile powder (Precipitins and IgG), significant decrease of DLCO, presence of ground glass opacities (only in subject #4), presence of alveolitis (in both) subjects with mild lymphocytosis (25% in subject #1). Four other patients with suggestive clinical symptoms occurring after argan handling but with no HRCT scan or DLCO changes were considered to present organic dust toxic syndrome, as a significant level of endotoxins (4200 EU/g) was observed in argan cake granulates. Finally, one of these patients presented a lobular emphysema on HRCT scan with altered DLCO, not modified by challenge to argan, and associated with active smoking.

The accuracy of the diagnosis of HP needs to be discussed. In the case index, the combination of symptoms, mild lymphocytosis on BAL, significant decrease of DLCO after challenge with complete recovery in the absence of exposure and the presence of specific serum antibodies with argan granulates or non-sterile powder, appeared to be sufficient to confirm the diagnosis of HP. In absence of lymphocytosis in the second case, the diagnosis of HP may be more doubtful. However, the presence of slight ground-glass opacities on HRCT scan, a significant decrease of DLCO after specific challenge, with recovery in the absence of exposure, as well as significant arcs to antigens derived from argan cake, were clearly in favor of the diagnosis. Moreover, all clinical features were mild and totally reversible after removal from occupational exposure, which could also explain the absence of respiratory crackles on physical examination, as also reported in 32% of patients with form one of the HP study. The positive diagnosis of HP is fairly complex [5] and several classifications have been proposed in the literature [4,16-18]. In most classifications, the major criterion is a combination of suggestive clinical symptoms after exposure to antigens, HRCT abnormalities, specific serum antibodies and lymphocytosis on BAL. However, a decrease in DLCO, positive challenge or inspiratory crackles are considered to be major or minor criteria depending on the classification, and these classifications remain controversial. In particular, a recent study observed that HRCT abnormalities were strongly associated with chronic forms of the disease, but may be absent in more acute forms. Accordingly, we assume that our two patients presented an acute form of HP (or active as proposed by Lacasse *et al.* in the HP study) [4]. Interestingly, the very low frequency of exposure (less than one hour every month for some workers) may explain the paucity and the transient nature of clinical features. The intensity of exposure and repeated exposure are known risk factors for HP [19-21]. The cases described here demonstrate that HP can also occur in very particular circumstances associated with infrequent exposure.

The marked differences between specific serum antibodies against non-sterile forms of argan (both granulate and powder) and sterile forms first suggest that a microbial agent may be responsible for the disease. However, cultures for the microorganisms usually involved in HP were negative, as were specific antibodies for these agents despite the use of several specific media. A review of the literature also found that argan roots are commonly contaminated by *Streptomyces marokkonensis* [10]. As some other *Streptomyces* spp. are known to be responsible for HP, this may constitute an interesting hypothesis to explain our findings. Unfortunately, all specific serological tests derived from the *Streptomyces marokkonensis* extract remained negative in all subjects. The last explanation, under the hypothesis of causative microbial agent, is that this agent is a no viable or uncultivable bacteria or fungi. However, these negative results and the fact that, to the best of our knowledge, HP has never been previously described in the literature among workers (mainly women) of argan cooperatives, also suggest that the antigen responsible could be a protein. In this case, differences observed between specific serum antibodies against argan granulates and powder and against the sterile form could be explained by a heat-labile protein. Further investigations are required to identify the protein or microbial agent responsible for the HP observed in these two patients.

## Conclusions

We report two cases of acute HP associated with argan powder manipulation before sterilization. No similar cases have been previously reported. Moreover, our findings also highlight the fact that HP can occur even in a context of infrequent exposure.

### Ethics

In this case series report, all the procedure are clinically indicated and confirmed by a pluridisciplicinary medical staff (multidisciplanarystaff for occupational allergic disease of the university hospital of Nancy). All cases agreed that theirs results could be published.

## Additional file

Additional file 1:
**Culture and antigen extract.**

## Abbreviations

BAL: Bronchoalveolar lavage
CO: Carbon monoxide
CD4: Cluster of differentiation 4
CD8: Cluster of differentiation 8
D0: Day zero (day of specific challenge)
D2: Day two (following specific challenge)
D4: Day four (following specific challenge)
DD: Ouchterlony double diffusion
DLCO: Carbon Monoxide Diffusing Capacity
ELISA: Enzyme-Linked ImmunoSorbent Assays
ES: Electrosyneresis on cellulose acetate
HRCT: High-resolution computed tomography
HP: Hypersensitivity pneumonitis
ODTS: Organic Dust Toxic Syndrome
