# Supplementary material for: Working with argan cake: a new etiology for hypersensitivity pneumonitis
Source: BMC Pulm Med. 2015 Mar 6;15:18. doi: 10.1186/s12890-015-0013-3 (PMC4369362; doi:10.1186/s12890-015-0013-3)
Supplement: Additional file 1: — Culture and antigen extract. [file 12890_2015_13_MOESM1_ESM.doc]

Supplementary data

Culture and isolation conditions.

One gram of each sample was washed with 20 mL of sterile distilled water containing 0.1% Tween 80 (Merck, Darmstadt, Germany) with a Stomacher® apparatus (AES Laboratory, Combourg, France). One hundred microliters of homogenized extracted sample were plated onto: 1) Mueller-Hinton agar (Mast Group Ltd., Bootle, Merseyside, England), 2) Dichloran 18% Glycerol (DG18) (Oxoid, Unipath, Basingstoke, UK) supplemented with 0.5% Chloramphenicol (Merck, Darmstadt, Germany) at 30°C 3) Difco actinomycetes isolation agar (BD Difco, Le Pont-De-Claix, France) 4) R8 medium according to Amner *et al*. . The two first media were incubated for 14 days at 20°C for isolation of non-fastidious bacteria and fungi; the last two media were incubated at 30°C and 52°C, respectively, to isolate mesophilic and thermophilic actinomycetes.

Antigen extract

Three total crude extracts were prepared by extraction from crude sample materials according to the form: granulates, powder and sterilized finished product. The native products was flatware of liquid of Coca (4 g sodium chloride, 2.75 g sodium bicarbonate, 4 g liquid phenol and completed to 1 L with sterile distilled water) and soaked for 7 days at room temperature while shaking at 300 r.p.m.. Several filtrations were performed on different filter papers and the last filtration was performed on a Millipore 0.45 µm filter. The filtrate was freeze-dried (Labconco, Kansas City, MB, USA). The antigen was reconstituted with sterile distilled water to a concentration of 100 mg/mL. This technique is adapted from Pépys *et al* .

Two somatic antigens were derived from bacteria (*Bacillus Licheniformis* and *Bacillus sp*.) isolated from “argan” (powder and granulated). The antigens were produced as previously described . Briefly, bacterial strains (*Bacillus licheniformis* and *Bacillus sp*.) were cultured on Mueller-Hinton medium for 1 week, sonicated, extracted overnight in NH_4_CO_3_ at 4°C, centrifuged at 13000 r.p.m., freeze-dried and standardized to 100 mg/mL of protein.

Antigen extracts for bacteria and fungal conidia for use by ELISA technique .

Bacteria and conidia were first suspended in a 10mM sodium phosphate 0.9% NaCl buffer pH=6 (0.09mM sodium phosphate monobase (Sigma-Aldrich®, St. Louis, Missouri), 0.01mM sodium hydrogeno phosphate dehydrate (Merck, Darmstadt, Germany), 0.9% NaCl (Sigma-Aldrich®) pH 6.0). For bacteria and conidia, 3ml of 10mM sodium phosphate 0.9% NaCl buffer were added on the culture, spread gently with a glass rake and aspirated. For each extract, 5 culture media were rinsed with this method, corresponding to 5 to 15ml of rinsing liquid available. After 5 min of centrifugation (Beckman Coulter, JA 20.1 rotor, 4°C, 6,000×g) to pellet bacteria and conidia, cells were suspended in 5ml of 0,1 M Tris-HCl-buffer at pH 8.5 supplemented with protease inhibitors (1mM of PMSF, 1mM of O-phenanthroline and 1mM of pepstatin (Sigma-Aldrich®)). The mixtures were incubated for 1 hour at room temperature with a recombinant β-1,3-glucanase (20 U/µL) (Lyticase, MP Biomedicals®, Irvine, California). During incubation, they underwent a continuous light movement that allowed the proteins on the surface to be released into the buffer. Soluble proteins were separated by two successive centrifugations (Beckman Coulter®, JA 20.1 rotor, 4°C, 3 min, 13,000×g). The supernatant was kept, then, 100µl of 0.1% deoxycholic acid solution (Sigma-Aldrich®) was added per millilitre and the preparation was incubated for 5 min at room temperature. Proteins were pelleted with 70µl of 0.6 M trichloroacetic acid (Sigma-Aldrich®) per millilitre of supernatant on ice, for 15 minutes. Precipitated proteins were subsequently pelleted by centrifugation (Beckman Coulter®, JA 20.1 rotor, 4°C, 15 min, 30,000×g). Protein extracts were purified with SDS-Page Clean-Up Kit (Roche Diagnostics®, Basel, Switzerland) as recommended by the manufacturer and suspended in an ELISA coating buffer. The protein concentration of each extract was determined by a protein dosage program (280nm) using the spectrophotometer ND-1000 Nanodrop™ (THERMO Fisher Scientific®, Waltham, Massachusetts).

References

1. Amner W, Edwards C, McCarthy AJ: **Improved medium for recovery and enumeration of the farmer's lung organism, Saccharomonospora viridis**. *Applied and environmental microbiology* 1989, **55**(10):2669-2674.

2. Pepys J, Jenkins PA, Festenstein GN, Gregory PH, Lacey ME, Skinner FA: **Farmer's lung: thermophilic actinomycetes as a source of "farmer's lung hay" antigen.** *Lancet* 1963, **2**(7308):607-611.

3. Reboux G, Piarroux R, Mauny F, Madroszyk A, Millon L, Bardonnet K, Dalphin JC: **Role of molds in farmer's lung disease in Eastern France**. *American journal of respiratory and critical care medicine* 2001, **163**(7):1534-1539.

4. Roussel S, Reboux G, Rognon B, Monod M, Grenouillet F, Quadroni M, Fellrath JM, Aubert JD, Dalphin JC, Millon L: **Comparison of three antigenic extracts of Eurotium amstelodami in serological diagnosis of farmer's lung disease**. *Clinical and vaccine immunology : CVI* 2010, **17**(1):160-167.
